# Bilateral tDCS on Primary Motor Cortex: Effects on Fast Arm Reaching Tasks

**DOI:** 10.1371/journal.pone.0160063

**Published:** 2016-08-04

**Authors:** Pablo Arias, Yoanna Corral-Bergantiños, Verónica Robles-García, Antonio Madrid, Antonio Oliviero, Javier Cudeiro

**Affiliations:** 1 Neuroscience and Motor Control Group (NEUROcom), Department of Medicine, INEF Galicia and Biomedical Research Institute of A Coruña (INIBIC), University of A Coruña, A Coruña, Spain; 2 FENNSI Group, Hospital Nacional de Parapléjicos, SESCAM, Toledo, Spain; 3 Centro de Estimulación Cerebral de Galicia, A Coruña, Spain; University of Bologna, ITALY

## Abstract

**Background:**

The effects produced by transcranial direct current stimulation (tDCS) applied to the motor system have been widely studied in the past, chiefly focused on primary motor cortex (M1) excitability. However, the effects on functional tasks are less well documented.

**Objective:**

This study aims to evaluate the effect of tDCS-M1 on goal-oriented actions (i.e., arm-reaching movements; ARM), in a reaction-time protocol.

**Methods:**

13 healthy subjects executed dominant ARM as fast as possible to one of two targets in front of them while surface EMG was recorded. Participants performed three different sessions. In each session they first executed ARM (*Pre)*, then received tDCS, and finally executed *Post*, similar to *Pre*. Subjects received three different types of tDCS, one per session: In one session the anode was on right-M1 (AR), and the cathode on the left-M1 (CL), thus termed *AR-CL*; *AL-CR* reversed the montage; and *Sham* session was applied likewise. Real stimulation was 1mA-10min while subjects at rest. Three different variables and their coefficients of variation (CV) were analyzed: Premotor times (PMT), reaction-times (RT) and movement-times (MT).

**Results:**

*triceps*-PMT were significantly increased at *Post*-*Sham*, suggesting fatigue. Results obtained with real tDCS were not different depending on the montage used, in both cases PMT were significantly reduced in all recorded muscles. RT and MT did not change for real or sham stimulation. RT-CV and PMT-CV were reduced after all stimulation protocols.

**Conclusion:**

tDCS reduces premotor time and fatigability during the execution of fast motor tasks. Possible underlying mechanisms are discussed.

## Introduction

Transcranial direct current stimulation (tDCS) is a promising tool for neurorehabilitation purposes. Over the past few years a solid background has been built on the capacity of tDCS to modulate functional brain networks and guidelines have been formulated for its safe use on humans [1, 2]. tDCS permits a transient modulation of cortical excitability by the application of currents in a non-invasive way. tDCS is able to produce long lasting depolarization or hyperpolarization of cell membranes depending on stimulation polarity [3]; these aftereffects are thought to be mediated by calcium-dependent plasticity of glutamatergic neurons [4]. As a result, anodal tDCS applied to the primary motor cortex (M1) increases cortico-spinal excitability and cathodal stimulation produces the opposite effect [3]. These effects depend on the intensity and duration of the stimulation [3], but just ten minutes at 1mA induces consistent aftereffects, lasting for a period of minutes [5, 6].

The classic electrode montage to modulate the excitability of the motor cortex places one electrode over M1 and the other at the contralateral supraorbital forehead [3]. However, simultaneous bilateral M1 stimulation induces a similar effect in terms of excitability modulation, weaker in magnitude than unilateral montages [7, 8], but with smaller inter-subject variability [7]. The use of bilateral tDCS-M1 is appealing in pathologies like stroke, where the interhemispheric imbalance might be controlled by up-regulating the excitability of the ipsilesional motor cortex, while down-regulating the contralesional M1 [9]. Also, it is believed that bilateral tDCS-M1 produces better effects than uni-hemispheric tDCS on behavioral motor tasks [10].

Despite what has been mentioned above, tDCS effects on functional motor tasks have received less attention than effects related to cortical excitability; however, its applicability seems suitable for rehabilitation and motor learning [11, 12]. Remarkably, tDCS of M1 modulates excitability at sites distant from the cortex [13], such as the propriospinal circuits within the spinal cord [14, 15]. This particular point should be considered when evaluating the effect of tDCS on goal-oriented motor tasks (including movement preparation and execution), because of a inter-play between M1 and spinal cord excitability precedes the execution of movements [16–18]. In fact, cathodal tDCS-M1 reduces cortical excitability but does not change spinal excitability [3, 5, 14], whereas anodal tDCS increases excitability in M1 [3, 6] but also modulates the excitability of propriospinal circuits [14, 15]. Therefore, it seems that the understanding of bilateral tDCS effects on motor functional activities is of relevance in order to design new strategies aimed to improve function in certain pathologies [19–21]. In this sense, one essential task in daily living activities is arm reaching.

A classical view of the parieto-frontal network considers several loci to study the effect of tDCS on arm reaching towards a target in the peri-personal space. The posterior parietal cortex receives input from visual areas through the dorsal stream [22] and generates representations of eye, head, body and surrounding frameworks for early visuomotor planning [23, 24] of reaching and grasping [22]; some of these functions are shared by M1 [25] and other areas [26]. The prefrontal cortex (PFC) has a role in the control of reaching, especially for choice reaching tasks [27, 28] in intimate relation with the dorsal premotor cortex (d)PMC [23, 29]; while the ventral premotor cortex (vPMC) may contribute to transformations from extrinsic to intrinsic coordinates to guide movement directed to objects in peri-personal space [23].

However, a modern view of this network is much wider, challenging the “serial” assumption that selection (decision making) occurs before specification (movement planning), and rather advocates that these processes operate simultaneously and in an integrated manner [30]. It is now suggested that potential reaching actions are specified by the dorsal stream, while their selection involves cortical (like PFC [30] or dPMC[31, 32]) and subcortical structures (like the Basal Ganglia [33, 34]) engaged in the evaluation of the opportunity of executing a reaching movement while minimizing costs and maximizing profits [35]. In this sense the network predicts future outcomes and cancels not sufficiently valuable actions [30]. Within the whole network M1 is a crucial area to study the effect of tDCS on reaching tasks for its role in planning and releasing the movement [23, 25, 35–38], as well on cancelling those that are on-going [32, 35]. M1 (amongst other areas) is also involved in choosing between alternative actions to get the goal and the generation of their motor commands [35, 39].

The effect of tDCS-M1 on the planning and execution of goal-oriented actions can be inferred from the reaction times (RT) and the movement times (MT), respectively [40]. In this study we have evaluated the effects of bilateral tDCS-M1 on arm reaching movements. We used RT tasks of different complexity while controlling the effect of polarity, and placebo.

Our research hypothesis is that tDCS modulates motor behavior in a polarity dependent manner. We predict faster responses after bilateral tDCS, when anodal stimulation is applied to the M1 contralateral to the dominant (executing) arm, as a result of the increased excitability of M1 circuits. Conversely, cathodal stimulation shall render opposite effects.

## Experimental Procedures

All experimental subjects signed consent forms. The protocol conformed to the declaration of Helsinki and was approved by the Ethics Committee of the University of A Coruña (Spain). The individuals whose experimental data were included in this manuscript have given written informed consent (as outlined in PLOS consent form) allowing the use of photographs to illustrate the figures.

### Subjects

Thirteen healthy subjects participated (six women, age range 20-37yrs). None took medication or undertook hard physical work in the week prior the experimental sessions. Subjects were right-handed [41] and had normal or corrected-to-normal vision.

### Procedure

**Fig 1:**
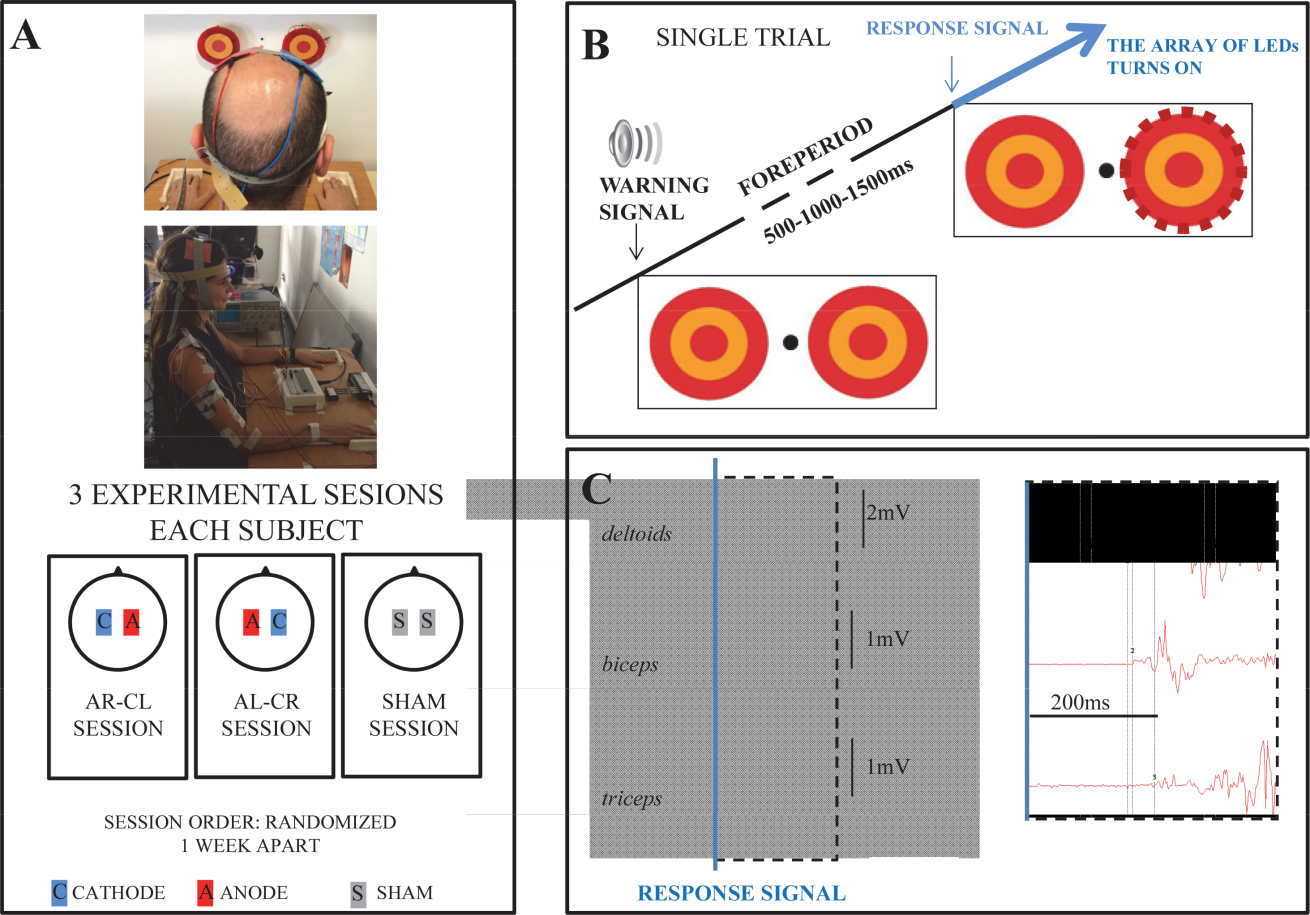
**(A)** Experimental setting and tDCS electrode montage in the 3 experimental sessions. The pictures show two subjects receiving tDCS at rest. **(B)** A single trial lasted 10s; the response signal was presented 500, 1000 or 1500ms after the warning cue. **(C)** Example of one recording reflecting the sequential activation of the three muscles evaluated. Recordings are synchronized to the response signal (marked as the blue vertical line). The dashed area is enlarged at the right to clarify the sequential muscle responses. The individuals in these pictures have given written informed consent (as outlined in PLOS consent form) to publish the images.

Each subject performed three sessions, one week apart. In each session they executed the reaching tasks before and after the tDCS (*Pre* and *Post*, respectively). A different tDCS protocol was applied in each session; the order was randomized. Subjects reached to one of two round targets placed at gaze height in front of them (see below). Reaching was always performed as fast as possible with the right (dominant) hand on a frontoparallel plane (Fig 1). No instructions were provided on how to touch the target, apart from asking subjects to touch the centre of the target with the hand as fast as possible. All subjects chose to touch with finger joints extended.

Each of the three sessions comprised three different reaching tasks randomized in order, and each task included several trials. In all cases a low-tone audible *warning signal* was presented as a cue prior to the *response-signal*. The turning-on of an array of red LED’s at the edge of the target was the *response-signal*. Without any other purpose than making the response-signal time unpredictable (with regards to the warning cue) we used three foreperiods (delays) between warning and response signals; 500, 1000, or 1500ms, and their presentation order was randomized within trials. The sequence of events was controlled by Signal-4 software via a CED 1401mkII (Cambridge Electronic Design, UK). Subjects were asked to fixate on a central point between the two targets until the appearance of the response-signal (Fig 1) after which they made the response.

#### Tasks

T1: Subjects were informed they had to reach the target ipsilateral to their dominant (executing) hand (i.e., *single-ipsilateral* response).

T2: Subjects were told that they had to reach the target in the contralateral space to their dominant arm (*single-contralateral*).

T3: In this task (*choice)* subjects were informed they would reach either the ipsilateral or the contralateral target to the dominant arm, depending on what target was lit (in randomized order).

Each of the two *single*-tasks (*single-ipsilateral* and *single-contralateral*) included 12 reaching trials, plus 1 catch trial, all randomized. In the catch trial the response signal did not appear after the warning cue.

The *choice*-task included 12 trials to the ipsilateral target (*choice-ipsilateral*) and 12 trials to the contralateral target (*choice-contralateral*) and these also included a catch trial. The 25 trials were randomized in order.

In all cases the inter-trial interval was 10s, and a one minute rest was given between the different tasks.

*Pre* and *Post* testing lasted 12min 10s each; *Post* started 1min after the end of the tDCS. The order of presentation of the three tasks in *Pre* was randomized, and reproduced in *Post*.

### Experimental Setting

The round targets were 15cm diameter; their centers were 32cm apart from each other, and halfway between them a small black spot (1 cm-diameter) served as the fixation spot. The subjects were seated on an adjustable chair, containing chest-straps to avoid trunk movements, but allowing unrestrained shoulder movements. The chair height was adapted to make eye level at fixation height; the distance from the subject to the targets was adjusted such that allowing the reaching to the contralateral target with nearly full-elbow extension, minimizing leg involvement in the task [42]. Subjects’ hands were on contact-plates. After setting, subjects made three fast ARM’s to each target, as practice. The entire setup (Fig 1) was reproduced in each of the different test days.

### Motor Outcomes

Signals were acquired by means of Biometrics-Data-Link, Digitimer D-360 amplifiers and the CED 1401 (3-3000Hz; 10KHz sampling rate, 1000 gain). Electromyographic (EMG) recordings determined the pre-motor time (PMT) [17], as the time lags from the *response-signal* to the movement-related EMG-onset [43]. EMG recorded the activity of the *deltoid*, *biceps brachii* and *triceps brachii*. Surface electrodes were placed in a belly-tendon montage on the *anterior deltoid*; *large biceps head*; and *lateral triceps head* (*del; bic; tri*), always after skin preparation. EMG-onset was determined automatically (and visually checked [44]) by applying the double-threshold method [45]. Thus the EMG signals were rectified and the EMG onset was considered at the first of ten consecutive samples (one threshold) above a given EMG amplitude (the other threshold); the latter threshold was equal to the mean EMG-background activity amplitude plus one standard deviation, which was calculated in the time-window just 50ms prior the response signal. Such threshold was obtained for each of the three muscles independently. The time elapsing from the LEDs flash to lifting the hand and leaving the contact-plate was computed as the RT; and the time from leaving the contact-plate to touch the target determined the MT. Customized MatLab programmes (The Mathworks, Ltd) were used to process the data.

### Brain Stimulation

tDCS was applied bilaterally on M1’s with a Neuroconn DC-Stimulator connected to a pair of 5x7cm saline-soaked electrodes. In all sessions, one electrode was placed on the left M1, and the other on the right M1; corresponding to C3 and C4 of the International 10–20 EEG system.

In one session the anode was on the left M1, and the cathode on the right M1, and this is referred as *AL-CR* montage. In other session the anode was on the right M1, and the cathode on the left M1 (*AR-CL*). *Sham* montage randomized electrode positions. For real stimulation 1mA-intensity was applied for 10min (current fade-in and out was ramped and lasted the initial and final 8s). The *Sham* protocol lasted the same time but the current was applied for 30s and then ceased [7]. Subject remained restful during the stimulation sessions.

### Statistical Methods

The mean of the responses for each experimental condition and subject was the outcome-value introduced in the analysis. The mean was computed considering all events from each experimental condition, but those which PMT <80ms or >800ms; thus those events with PMT <80ms were considered anticipations, and their proportion in the different stimulation protocols, conditions and testing time-points (*pre* and *post*) were evaluated with the Fisher Exact Probability Test. The threshold of 80ms was set based on the latencies of a visual-evoked potential to a flashing LED recorded in the primary visual cortex (V1), plus the minimum latency for an interaction between V1 and M1, plus the latency from M1 activation to EMG onset on the studied muscles [46–48]. Events with PMT >800ms were discarded as sign of un-attentiveness [49], though this happened just once in all subjects and conditions. PMT, RT and MT from events within those thresholds were normalized to control variability related to daily differences in the experimental setting (see above), though care was taken to minimize it. Normalization was performed as follows: For each session the average for each variable was calculated from data including *Pre* outcome-values for all tasks and subjects (Table 1). This normalizing value was used to divide all subjects’ responses at *Pre* and *Post* for the corresponding day. This normalization procedure respects inter-subject variability, while normalizing the responses to the daily pooled *Pre*-testing.

**Table 1:** PMT, RT and MT at *Pre* in the three days of the protocol; mean (SE) considering all subjects.

	*AL-CR*	*AR-CL*	*Sham*
**PMT (ms) at *Pre***	201.8 (11.5)	204.5ms (12.3)	201.0ms (10.5)
**RT (ms) at *Pre***	243.3 (9.0)	245.1ms (10.0)	245.1ms (9.0)
**MT(ms) at *Pre***	222.5 (12.5)	227.0ms (10.3)	221.0ms (12.0)

After checking the normality of the distributions with a Kolgomorov-Smirnov test for one sample, an ANOVA with repeated measures analyzed the effect of tDCS on the variables considering the 13 subjects.

We have used two different ANOVA designs: one for the PMT and one for RT and MT. The former is a five factors ANOVA with STIM (three levels: *AR-CL*, *AL-CR*, and *Sham*); Time (two levels: *Pre* and *Post*); LATERALITY (two levels: Target *Ipsilateral* or *Contralateral* to the dominant–executing- hand); OPTION (two levels: *single* and *choice* responses) and MUSCLE (three levels: *deltoid*, *biceps* and *triceps*). The ANOVA on RT and MT had the same design except for the factor MUSCLE which was not included since RT and MT derived from contact plates.

The W-Mauchly test checked the sphericity for ANOVA, if sphericity was violated the ANOVA degrees of freedom were corrected by means of the Greenhouse-Geisser coefficients. Effect sides were calculated by partial etha and etha squared (η_p_^2^, η^2^). Significance was considered if p<0.05.

## Results

Table 1 shows the mean PMT, RT, and MT at *Pre* under each experimental condition (values serving as normalizing factors and equivalent to the units in the y-axes of the corresponding graph). Subjects made no responses during the catch trials. See also S1 Data.

### Effects of Brain Stimulation on PMT

Table 2 shows the mean values for the different levels of all factors in a *pre-post* basis.

**Table 2:** Mean values in ms (and SE) for the different levels of the different factors in a *pre-post* basis. PMT: premotor times; RT: reaction times; MT: movement times.

	*AL-CR*		*AR-CL*		*Sham*	
	**TIME**		**TIME**		**TIME**	
**PMT**	***pre***	***post***	***pre***	***post***	***pre***	***post***
**LATERALITY**						
***ipsi***	198.7 (12.9)	194.8 (9.9)	203.8 (11.1)	204.4 (14.1)	198.5 (11.3)	195.9 (10.4)
***contra***	204.9 (10.6)	197.7 (9.3)	205.2 (13.8)	204.3 (14.5)	203.5 (9.8)	208.1 (10.0)
**OPTION**						
***single***	194.6 (12.8)	190.7 (9.7)	196.0 (10.6)	196.2 (14.1)	193.0 (11.1)	195.5 (10.3)
***choice***	209.0 (11.2)	201.8 (9.6)	213.0 (14.4)	212.5 (14.8)	209.0 (10.5)	208.6 (10.5)
**MUSCLE**						
***del***	191.4 (9.9)	184.9 (7.8)	190.0 (10.2)	190.6 (12.2)	192.2 (8.7)	189.7 (8.4)
***bic***	188.4 (10.8)	183.9 (9.0)	187.5 (13.0)	186.0 (14.0)	189.6 (9.9)	187.1 (9.5)
***tri***	225.6 (16.0)	220.0 (13.7)	236.0 (16.9)	236.5 (19.2)	221.1 (14.4)	229.2 (14.8)
**RT**	***Pre***	***post***	***pre***	***post***	***pre***	***post***
**LATERALITY**						
***ipsi***	241.4 (9.9)	241.2 (6.9)	243.7 (8.3)	243.1 (11.2)	241.9 (9.4)	241.5 (9.3)
***contra***	245.2 (8.4)	246.3 (8.6)	246.5 (12.0)	243.2 (11.6)	248.3 (8.8)	255.1 (11.2)
**OPTION**						
***single***	237.0 (9.8)	238.1 (8.7)	237.2 (9.3)	236.0 (11.6)	238.6 (10.9)	240.7 (11.0)
***choice***	249.6 (9.3)	249.3 (6.7)	253.0 (11.3)	250.3 (11.5)	251.6 (8.2)	255.8 (9.2)
**MT**	***pre***	***post***	***pre***	***post***	***pre***	***post***
**LATERALITY**						
***ipsi***	205.4 (11.0)	207.0 (11.9)	206.8 (8.7)	202.2 (8.8)	203.3 (10.8)	204.8 (9.6)
***contra***	239.6 (14.2)	242.4 (16.9)	247.2 (12.2)	242.6 (12.5)	238.7 (13.6)	236.8 (13.0)
**OPTION**						
***single***	220.7 (12.2)	222.8 (14.1)	229.1 (9.2)	221.7 (10.0)	218.7 (11.3)	221.1 (11.4)
***choice***	224.3 (12.9)	226.6 (14.5)	224.9 (11.6)	223.1 (11.2)	223.3 (12.8)	220.4 (11.2)

*Ipsi*, *contra*: responses to the ipsilateral or contralateral targets to the dominant-executing hand. *Del* (deltoids), *bic* (biceps), *tri* (triceps).

Table 3 summarizes the effects of tDCS on PMT (ANOVA factors, and significant interactions of the factors with TIME). If interactions TIME x STIM were significant (first column of results in the table), we followed-up with ANOVA’s by pair of STIM modes and, if significance remained, by STIM mode in isolation (subsequent columns in Table 3).

**Table 3:** ANOVA’s for PMT. Main effects and significant interactions with factor TIME. ANOVA’s were executed considering the three STIM modes. If significant interactions indicate different responses to STIM in the testing TIMEs, the ANOVA’s were followed-up by pairs and the by single STIM mode.

	*AR-CL vs AL-CR vs Sham*	*AR-CL vs Sham*	*AL-CR vs Sham*	*AR-CL vs AL-CR*	*Sham*
**MAIN EFFECTS**					
STIM	F_2,24_ = 0.3 p = 0.3	F_1,12_ = 0.1 p = 0.9	F_1,12_ = 0.5 p = 0.5	F_1,12_ = 0.4 p = 0.5	N.A
TIME	F_1,12_ = 2.4 p = 0.15	F_1,12_ = 0.8 p = 0.8	F_1,12_ = 1.4 p = 0.3	F_1,12_ = 4.9 **p = 0.048**	F_1,12_ = 0.2 p = 0.6
				η_p_^2^ = 0.288	
OPTION	F_1,12_ = 24.6 **p<0.001**	F_1,12_ = 30.3 **p<0.001**	F_1,12_ = 16.5 **p = 0.002**	F_1,12_ = 19.7 **p<0.001**	F_1,12_ = 16.0 **p = 0.002**
	η_p_^2^ = 0.672	η_p_^2^ = 0.716	η_p_^2^ = 0.579	η_p_^2^ = 0.621	η_p_^2^ = 0.572
LATERALITY	F_1,12_ = 7.0 **p = 0.022**	F_1,12_ = 6.4 **p = 0.027**	F_1,12_ = 6.6 **p = 0.025**	F_1,12_ = 2.2 p = 0.2	F_1,12_ = 16.2 **p = 0.002**
	η_p_^2^ = 0.368	η_p_^2^ = 0.347	η_p_^2^ = 0.355		η_p_^2^ = 0.574
MUSCLE	F_2,24_ = 30.3_Ԑ = 0.6_ **p<0.001**	F_2,24_ = 31.3_Ԑ = 0.6_ **p<0.001**	F_2,24_ = 22.6_Ԑ = 0.6_ **p<0.001**	F_2,24_ = 30.5_Ԑ = 0.6_ **p<0.001**	F_2,24_ = 21.5_Ԑ = 0.6_ **p<0.001**
	η_p_^2^ = 0.876	η_p_^2^ = 0.723	η_p_^2^ = 0.653	η_p_^2^ = 0.717	η_p_^2^ = 0.642
**SIGNIFICANT**	F_2,24_ = 3.4 p = 0.052_STIMxTIMExLAT_	F_1,12_ = 5.6 **p = 0.035**_STIMxTIMExLAT_	F_1,12_ = 6.1 **p = 0.030**_STIMxTIMExLAT_	N.S	F_1,12_ = 11.4 **p = 0.005**_TIMExLAT_
**INTERACTIONS**	F_4,48_ = 3.2 **p = 0.020**_STIMxTIMExMUS_	F_2,24_ = 6.8 **p = 0.042**_STIMxTIMExOPT_	F_2,24_ = 5.4_Ԑ = 0.7_ **p = 0.024**_STIMxTIMExMUS_		F_2,24_ = 9.5 _Ԑ = 0.6_ **p = 0.005**_TIMExMUS_
**FACTOR TIME**			F_1,12_ = 5.7 **p = 0.035**_STIMxTIME_		

N.S. = none was significant; N.A. = not applicable since such ANOVA had not that factor. Partial etha squared (η_p_^2^) is reported for significant main effects. Since significant interactions involving TIME and STIM (in the model with 3 STIM modes) do not inform whether the three STIM modes produced different responses compared to each other, or if there was just one STIM mode that produced different responses in TIME compared to the other two STIM modes, we followed-up ANOVA by pairs of STIM modes, and if needed, just with one STIM mode.

With the 3 STIM modes-ANOVA there were significant main effects showing that all PMT were faster in single compared to choice responses (η_p_^2^ = 0.672; η^2^ = 0.061), in ipsilateral than contralteral responses (η_p_^2^ = 0.368; η^2^ = 0.006); and presented a sequential muscle activation (η_p_^2^ = 0.716 η^2^ = 0.393; the latter can be observed in a representative subject in Fig 1C).

**Fig 2:**
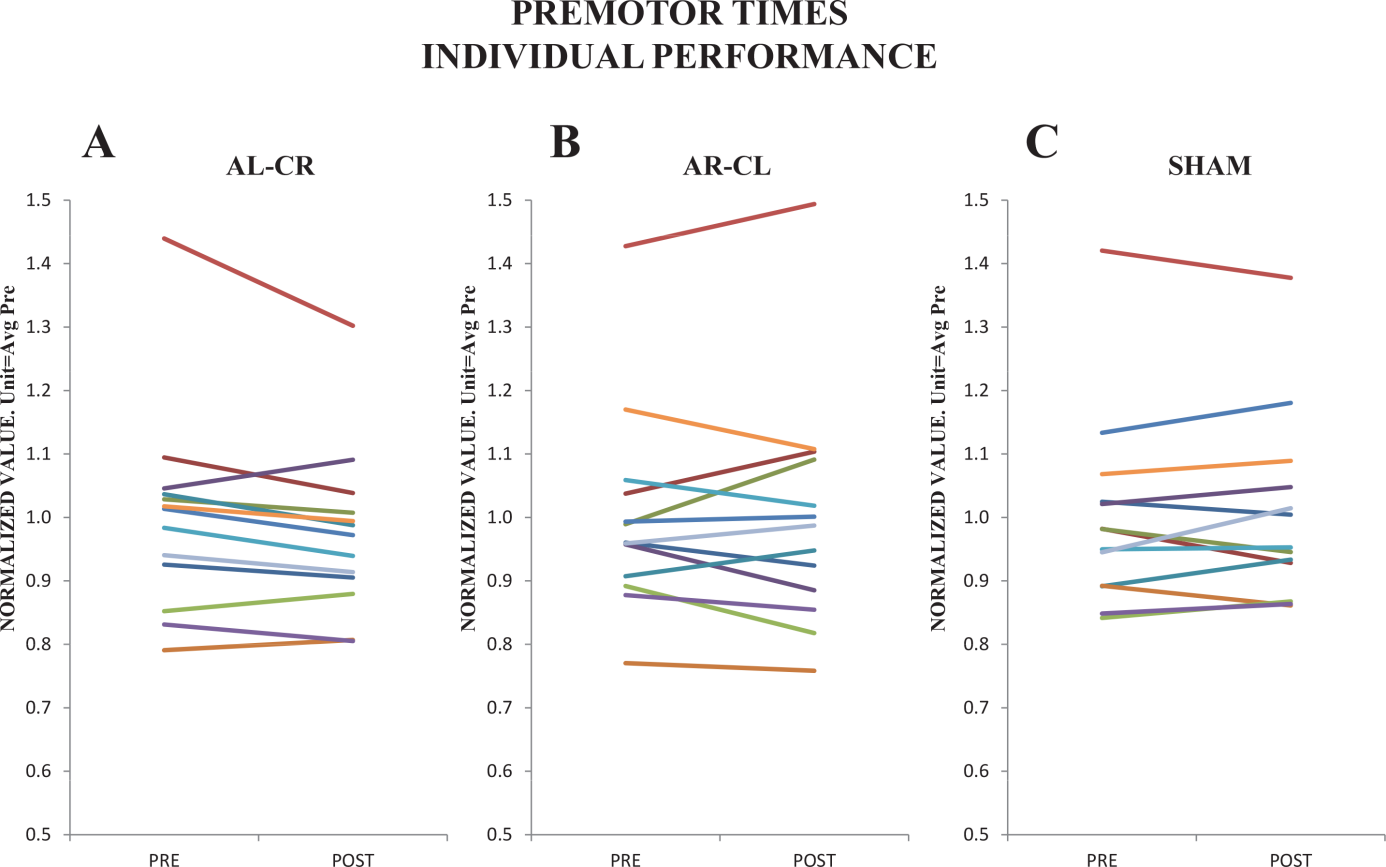
Individuals’ responses for PMT. The *y*-axis unit indicates the mean response considering all subjects and conditions at *Pre*. It was equivalent to 201.8ms (*sem* 11.5) for AL-CR **(A)**; 204.5ms (*sem* 12.3) for AR-CL **(B)**; and 201.0ms (*sem* 10.5) for Sham sessions **(C)**.

The significant interactions between TIME and STIM with other factors were observed in this model with 3 stimulation modes (also in the rest of models, except when the two active tDCS protocols were compared), Table 3. This means that the responses to Sham were different to the responses of the other to stimulation modes, and that the responses obtained with the two active stimulation modes were not significantly different of each other; for such reason their effects are shown pooled in figures (green tones). The individual’s responses in *Pre* vs. *Post* basis are depicted in Fig 2.

**Fig 3:**
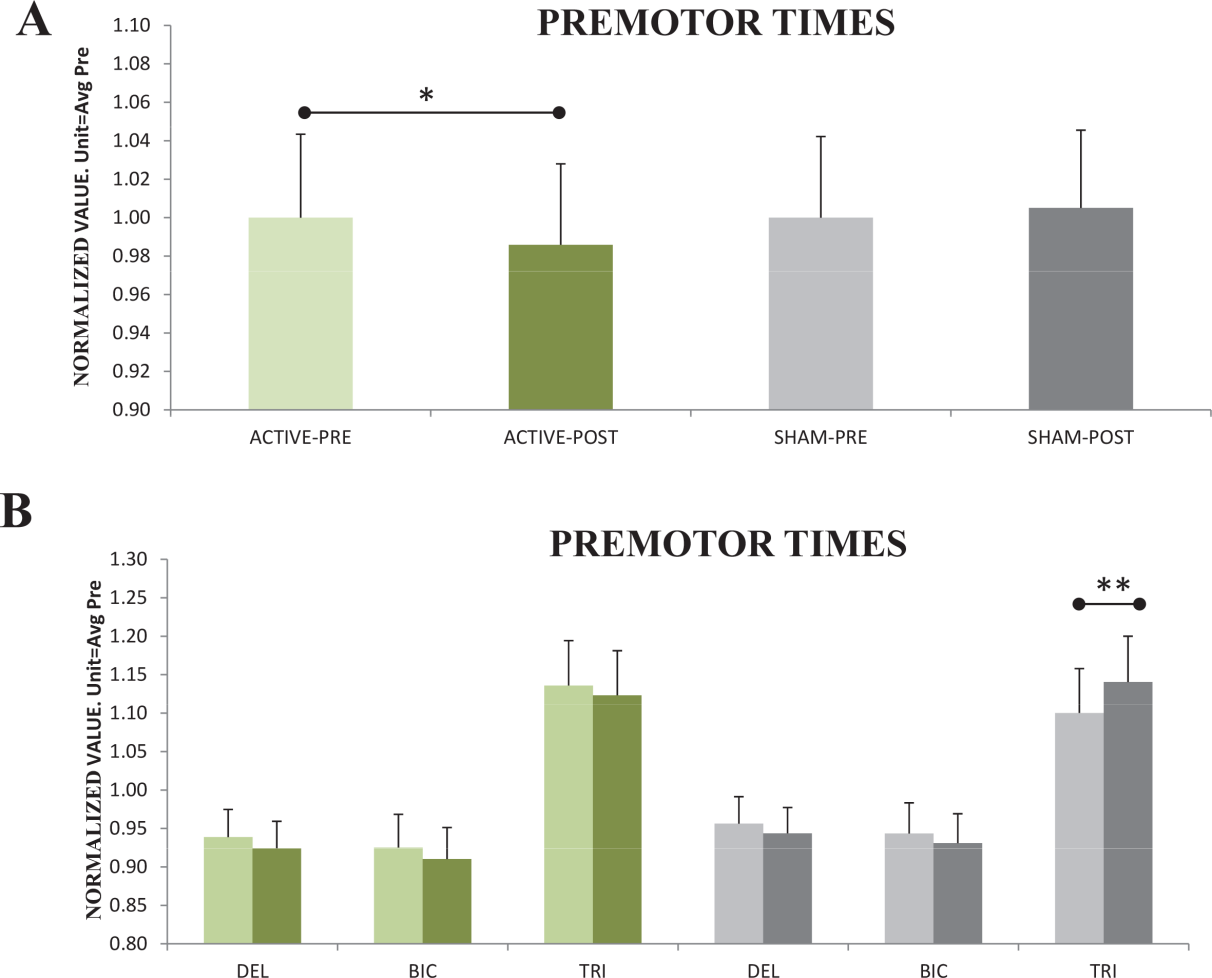
**(A)** PMT at *Post* were differently modulated by *Sham*-tDCS compared to the other two active protocols, which did not differ each other (shown pooled in green tones). There was a significant decrease at *Post* after both active protocols. **(B)** Sham stimulation increased *Post* PMT, specifically in the *triceps* muscle. The *y*-axis unit indicates the mean response across all subjects and conditions at *Pre*. *p<0.05; **p<0.*01*.

For the follow-up ANOVA including *AR-CL* and *AL-CR* stim modes the significant factor TIME, in absence of significant interactions with any other factor, indicates a small (η_p_^2^ = 0.288 η^2^ = 0.002) but significant (F_1,12_ = 4.9 p<0.05_TIME_) reduction of 1.5% in the PMT at *Post* (Fig 3A). This effect was present in the three studied muscles and was independent on the laterality and options of the responses. Fig 2 indicates that in most of the subjects the reduction in the PMT was small, which explains the small (although significant) effect observed.

For the ANOVA with Sham stimulation, the effects were rather the opposite. The effect of Sham stimulation considering the three muscle together was a very mild (surely non significant) increase of PMT in the Post condition (Fig 3A). However the Sham effects where different for the three muscles (F_2,24_ = 9.5_Ԑ = 0.6_ p = 0.005_TIMExMUS_ η_p_^2^ = 0.443 η^2^ = 0.009), and PMT increased significantly (4%) at *Post* in the *triceps* (*post-hoc* p = 0.002) (Fig 3B).

### Effects of Brain Stimulation on RT and MT

Table 2 shows the mean values for the different levels of the different factors in a *pre-post* basis, in the RT and MT.

Table 4 shows significant main effects, indicating that the responses were faster in the case of the single than in choice tasks for RT (η_p_^2^ = 0.659; η^2^ = 0.069). On the other hand, ipsilateral were faster than contralateral responses, this was shown by a significant main effect of factor “laterality” for RT (η_p_^2^ = 0.291; η^2^ = 0.010) and also for MT (η_p_^2^ = 0.873; η^2^ = 0.495).

**Table 4:** ANOVA’s for variables which response was not different for the three stimulation modes.

						SIGNIFICANT
*AR-CL vs AL-CR vs Sham*	MAIN EFFECTS					INTERACTIONS
						FACTOR TIME
	STIM MODE	TIME	OPTION	LATERALITY	MUSCLE	
**RT**	F_2,24_ = 0.2 p = 0.9	F_1,12_ = 0.1 p = 0.8	F_1,12_ = 23.2 **p<0.001**	F_1,12_ = 4.9 **p = 0.046**	N.A	N.S
			η_p_^2^ = 0.659	η_p_^2^ = 0.291		
**MT**	F_2,24_ = 0.1 p = 0.9	F_1,12_ = 0.3 p = 0.6	F_1,12_ = 1.3 p = 0.3	F_1,12_ = 82.2 **p<0.001**	N.A	N.S
				η_p_^2^ = 0.873		
**CV-PMT**	F_2,24_ = 0.7 p = 0.5	F_1,12_ = 25.1 **p<0.001**	F_1,12_ = 1.3 p = 0.3	F_1,12_ = 1.5 p = 0.2	F_2,24_ = 0.1 p = 0.4	N.S
		η_p_^2^ = 0.677				
**CV-RT**	F_2,24_ = 0.6 p = 0.6	F_1,12_ = 7.1 **p = 0.020**	F_1,12_ = 0.3 p = 0.6	F_1,12_ = 1.9 p = 0.2	N.A	N.S
		η_p_^2^ = 0.373				
**CV-MT**	F_2,24_ = 1.4 p = 0.3	F_1,12_ = 0.7 p = 0.4	F_1,12_ = 0.1 p = 0.7	F_1,12_ = 3.1 p = 0.1	N.A	N.S

N.A = not applicable since RT, MT and their CV’s were not obtained from EMG but from contact plates. N.S = none was significant. Partial etha squared (η_p_^2^) is reported for significant main effects.

Table 4 also shows that RT and MT were not modified by the different stimulation modes. For both variables factor TIME was never significant and it did not interact significantly with any other factor.

### Effects of Brain Stimulation on CV’s of PMT, RT and MT

**Fig 4:**
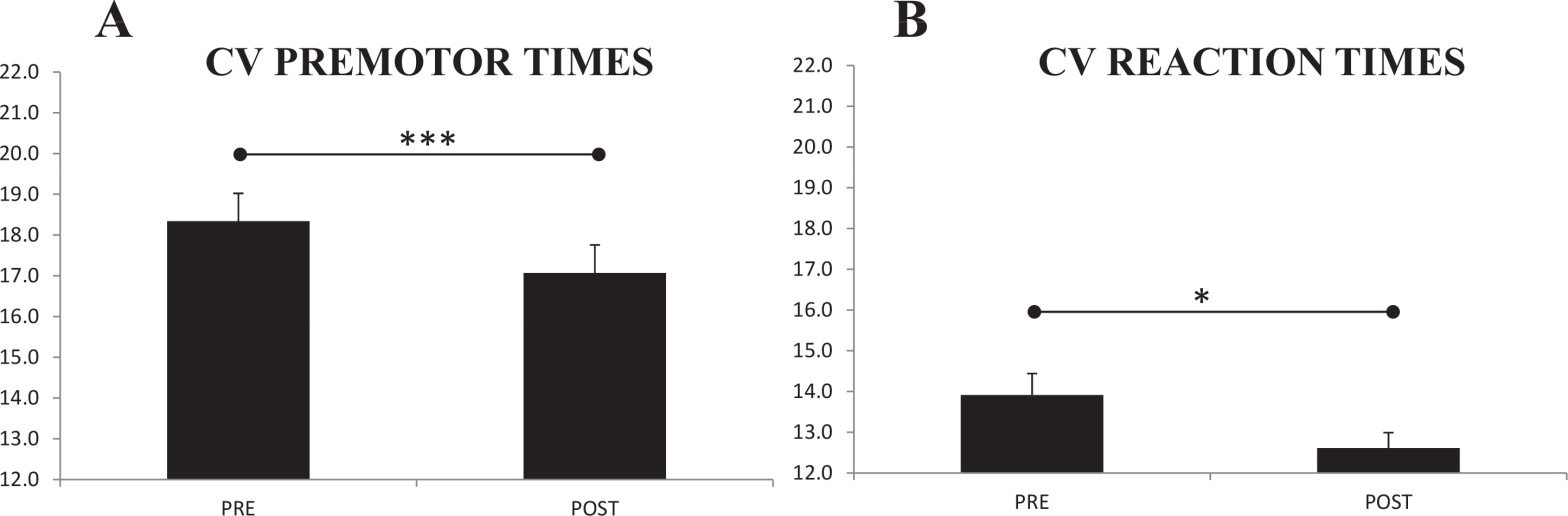
**(A)** The CV of PMT was reduced significantly at *Post*, regardless stimulation modes, tasks or muscles (so that shown pooled). **(B)** A similar pattern was found for the CV of RT. *p<0.05; ***p<0.*001*.

Table 4 indicates a change in the CV’s of PMT and RT at *post* compared to *pre*; but not in the CV of MT. Since there was a significant main effect of factor “time” for CV-PMT and for CV-RT (Fig 4A and 4B respectively), but there were not significant interactions with any other factor, this means that the significant reductions in the CV’s after tDCS (from 18.3 to 17.0% in PMT; and from 13.9 to 12.6% in RT) were observed in all tasks (and muscles for PMT); and also in all stimulation protocols, including Sham (η_p_^2^ = 0.677, η^2^ = 0.01 for PMT; and η_p_^2^ = 0.373, η^2^ = 0.014 for RT).

### Effects of Brain Stimulation on Anticipatory Responses

A total of 70 reaching movements were anticipatory responses, this is 5.6% of the 1,248 movements executed, in all subjects. The proportion of anticipations at Pre and Post was not significant different (Fisher Exact Probability Test p>0.05). Fragmentation of this analysis for the different tasks at Pre and Post was not considered due to the reduced number of anticipations.

## Discussion

In the tasks performed in our experiments we have observed some well known features of reaction responses: i.) faster reactions to single than to choice options [50, 51]; ii.) faster reactions with ipsilateral than contralateral movements [52] and iii.) sequential muscle activation during reaching tasks [53–55]. However, the main finding of this work is that real tDCS of M1 reduces the PMT of reaching movements. Remarkably, the effect of tDCS was not different for the two active electrode montages. The significant increase in triceps PMT *Post*-*Sham* might be explained by fatigability, since the muscle has a main role in the fast projection of the hand towards the target. Fatigability (i.e., reduction of performance at *Post*) was not only avoided by real stimulation, but the net effect was a step further and reduced PMT (in all muscles). Admittedly the reduction of PMT after active tDCS was small but statistically significant.

### Sham Effects on PMT and RT

The increment in PMT after *Sham* stimulation was localized at the level of the *triceps* muscle, without affecting the *deltoids* and *biceps*. These results might provide some insights on the operational mechanisms at *post-Sham*. The role of the *triceps* is of capital importance in the projection of the hand to the target, as an elbow extensor, while, on the other hand, the *biceps* is an elbow and shoulder flexor, this latter function shared with *anterior head of deltoids*. Thus, *deltoids* and *biceps* main involvement in the tasks is to lift the hand from the plates; later on the *triceps* is activated to project the hand towards the target [54]. Such a sequential activation is reflected in all our EMG recordings. It is tempting to speculate that, in our protocol, fatigue only affects the more demanding muscular activity and, consequently, only the triceps was affected post *Sham* (active stimulation affected all muscles, see discussion below). These results matched the fact that RT (recorded at plate lift) were unaffected by the *Sham* stimulation. Therefore task progression might induce fatigue which is reflected in an alteration in muscle recruitment patterns.

Remarkably fatigue might arise from different sources. Muscle fatigue is defined as a progressive failure of muscle-output generating capacity during and after the tasks. Its origin appears to lie in a deficit of the neural motor system to generate or propagate the action potentials to the muscle in an efficient manner [56]—this is a form of central fatigue (CF). However, CF might also emerge from other (non motor) factors (i.e., mental fatigue) [57, 58] where the increase in PMT during a prolonged period of attention might be a marker of cognitive functionality waning [57, 58]. Bilateral tDCS of prefrontal areas (regardless polarity) has been shown to improve cognitive processes like attention [59], and it seems that tDCS improved attention might be a key element to reduce high order expressions of fatigability (such as mental fatigue) [57].

Despite that with our protocol we cannot discard that the reduction in PMT after bilateral tDCS could be related to modulations of cognitive processing, we believe that mental fatigue expression on PMT should have been shown in all the three muscles recorded, and not only in the *triceps*. Note also that the anticipatory responses (likely to be related with mental fatigue) were not modified by the protocols, and that very delayed PMT reflecting loss of attention (longer than 800ms) were present only once across subjects, which weakens the possibility that the effects of our tDCS protocol are related to cognitive processing.

### Active tDCS Reduces PMT Regardless Polarity

Contrary to our initial hypothesis, the effects observed with real stimulation were polarity independent. Our hypothesis followed the “*contralaterality of motor control*”[60]; this considers the presence of inter-hemispheric interactions between both motor areas, with a stronger inhibition on the non-dominant M1 emerging from the dominant motor cortex [60–64]. Considering such asymmetry, it should never be expected that both active tDCS montages produce a same effect. However, it should also be considered that there are a number of ipsilateral projections from the motor areas to the spinal cord [65]. These ipsilateral projections likely target a great proportion of motoneuron pools controlling proximal limb muscles (like those evaluated in our study *deltoids*, *biceps* or *triceps*) [66], which might contribute to our polarity independent results. In fact, there is a growing amount of data showing that when we move a hand or an arm the activity in both the contralateral and the ipsilateral hemispheres are simultaneously activated. The neurophysiological significance of the bilateral activation of the motor cortices remains unclear. Kobayashi et al., [64] suggested that “ipsilateral activation during non-dominant hand movements could reflect an increased inhibition exerted by the right over the left hemisphere through callosal fibers”. Others support the idea that all movements are initiated in the dominant hemisphere with the non-dominant would be responsible just for the execution of the command issued by the dominant hemisphere [60]. Interestingly, we started with an hypothesis which would sustain in principle the theory of contralaterality of motor control (i.e. “we predict a shortening of the MT and RT after bilateral tDCS, when anodal stimulation is applied to the M1 contralateral to the executing arm, as a result of the increased excitability of M1 circuits”), but we ended up with something completely different which seem to follow the new stream of thought.

### Effects of tDCS on RT and MT

Our results indicate that active tDCS reduces PMT but not RT. At a first glance these results seems incompatible. Reynolds and Ashby [67] have shown that PMT are periods characterized by a progressive reduction of intracortical inhibition. This is to say that the level of intracortical inhibition is minimal from the last ≈80ms preceding EMG-onset to end of the PMT [67], as well as during muscle contraction [68]. As mentioned before, this inhibitory process is likely to be modulated by tDCS [69]. However, RT (once finished PMT) corresponds with periods where inhibition is not altered much and, perhaps not affected by tDCS.

Likewise, MT were unaffected by tDCS. Since the reaction-time protocols reflect both, cognitive and motor components, the lack of changes in MT seems to favor a specific effect of tDCS on the cognitive component of the response. However, some evidences might not support this possibility. Firstly, PMT was differently affected in the three muscles after Sham. This suggests an alteration in muscle recruitment rather than in cognitive processing. Second, some previous work has indicated that MT variations depend on the speed of reactions; faster reactions produce increments in MT and vice versa [33, 52]; thus, perhaps the option that tDCS prevents the increment of MT should be taken into account.

### Effects of tDCS on Variability of Responses

Some previous work indicates that tDCS on parietal cortex reduces the variability of time estimation if applied to the left hemisphere, or impacts the accuracy of time estimation after right hemisphere stimulation [70]. However, in our case, the reduction of the variability (in PMT and RT, but not of MT) occurred after all stimulation protocols, either active or *Sham*. The reduction of variability during repetitive reaching movements is not a new finding and has been related to progressive appearance of fatigue along the task. In this situation the kinematics of reaching movements becomes more stereotyped [71] and movements less variable. However, we believe that a reduction of CV’s of PMT and RT simply due to motor practice is also possible. The protocol was not conceived to test this hypothesis but this is a possibility to be considered since the reduction in CV was observed in all muscles (regardless they expressed fatigue -i.e., *triceps*- post Sham or not -i.e., *deltoids* and *biceps*-) and in all stimulation conditions. Though this possibility does not explain why the CV of MT was not reduced with practice, it is plausible that different expressions of a motor act might need different levels of practice to reduce their variability.

### Limitations of the Study

Our study only explored the effects of tDCS on the dominant limb. It is possible that effect sizes would have been greater in the case of studying the non-dominant hand, because in healthy subjects motor execution with the dominant arm is likely to experience ceiling-effects. In fact, some reports indicate greater effects of tDCS for the non-dominant limb [72]. In addition our sample size is modest, therefore future studies should clarify the effect of tDCS on reaching movements executed with dominant/non-dominant arms, on greater samples.

## Conclusions

In our hands, bilateral tDCS-M1 reduced PMT and avoided fatigability in a functional reaching task; the effects were polarity independent. Futures studies might include different reaction time protocols to disentangle the cognitive and motor effects produced by tDCS on these kinds of tasks.

## Supporting Information

S1 Data Data Set. (RAR)
