# Correction: Bilateral tDCS on Primary Motor Cortex: Effects on Fast Arm Reaching Tasks

**DOI:** 10.1371/journal.pone.0163714

**Published:** 2016-09-22

**Authors:** The PLOS ONE Staff

Fig 1C was incorrectly reproduced during typesetting. Please view the corrected Fig 1 here. The publisher apologizes for this error.

**Fig 1:**
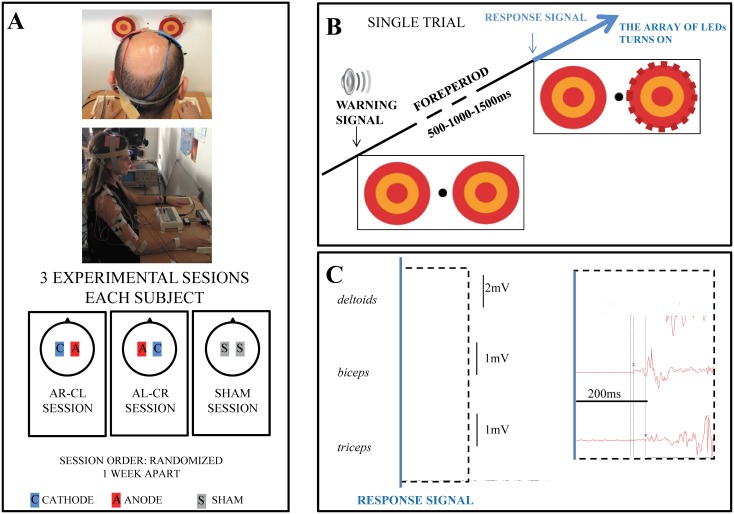
**(A)** Experimental setting and tDCS electrode montage in the 3 experimental sessions. The pictures show two subjects receiving tDCS at rest. **(B)** A single trial lasted 10s; the response signal was presented 500, 1000 or 1500ms after the warning cue. **(C)** Example of one recording reflecting the sequential activation of the three muscles evaluated. Recordings are synchronized to the response signal (marked as the blue vertical line). The dashed area is enlarged at the right to clarify the sequential muscle responses. The individuals in these pictures have given written informed consent (as outlined in PLOS consent form) to publish the images
